# Supplementary material for: Silica deposits on Mars with features resembling hot spring biosignatures at El Tatio in Chile
Source: Nat Commun. 2016 Nov 17;7:13554. doi: 10.1038/ncomms13554 (PMC5473637; doi:10.1038/ncomms13554)
Supplement: Supplementary Information — Supplementary Figures 1-5 and Supplementary Table 1. [file ncomms13554-s1.pdf]

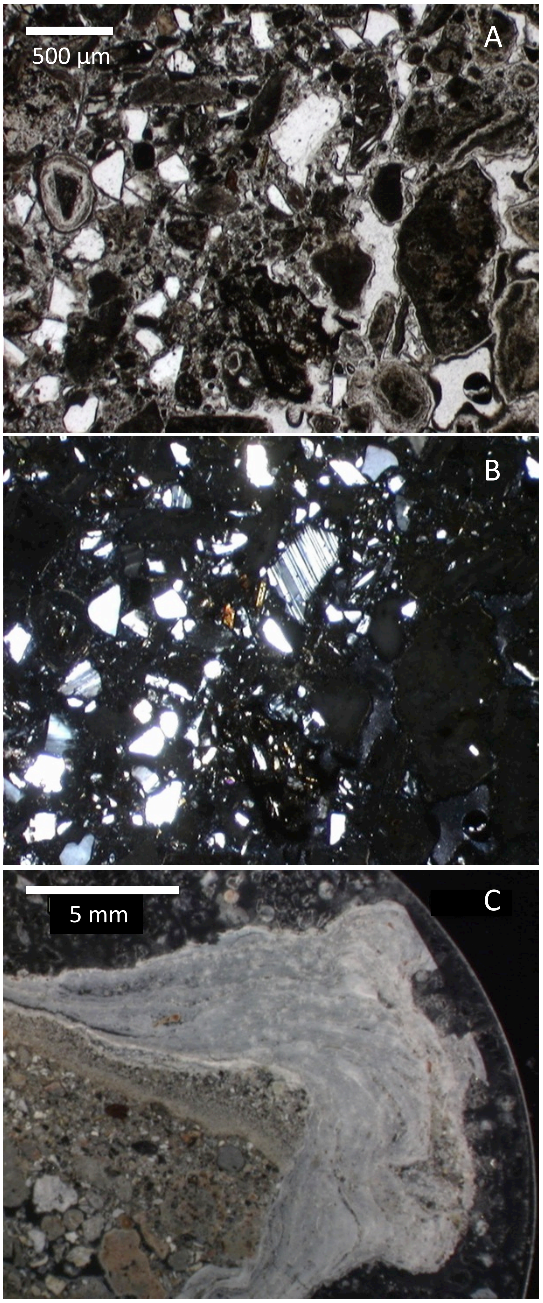

Supplementary Figure 1 | Microscopic views of El Tatio sinter breccia ET1-1C. (A) Transmitted plane light and **(B)** crossed polarized light thin section photomicrographs showing a variety of glassy to finely porphyritic volcanic rocks, quartz and feldspar grains, and coated grains and fragments of older siliceous sinter, lightly cemented by opaline silica (darkened areas in **B**). **(C)** Reflected light photo of a one-inch round thin section showing the accretion profile of a siliceous digitate structure that grew on a pebble of breccia in a discharge channel. The pebble is composed of locally derived and transported volcaniclastic sediments that were consolidated by silica cementation following deposition.

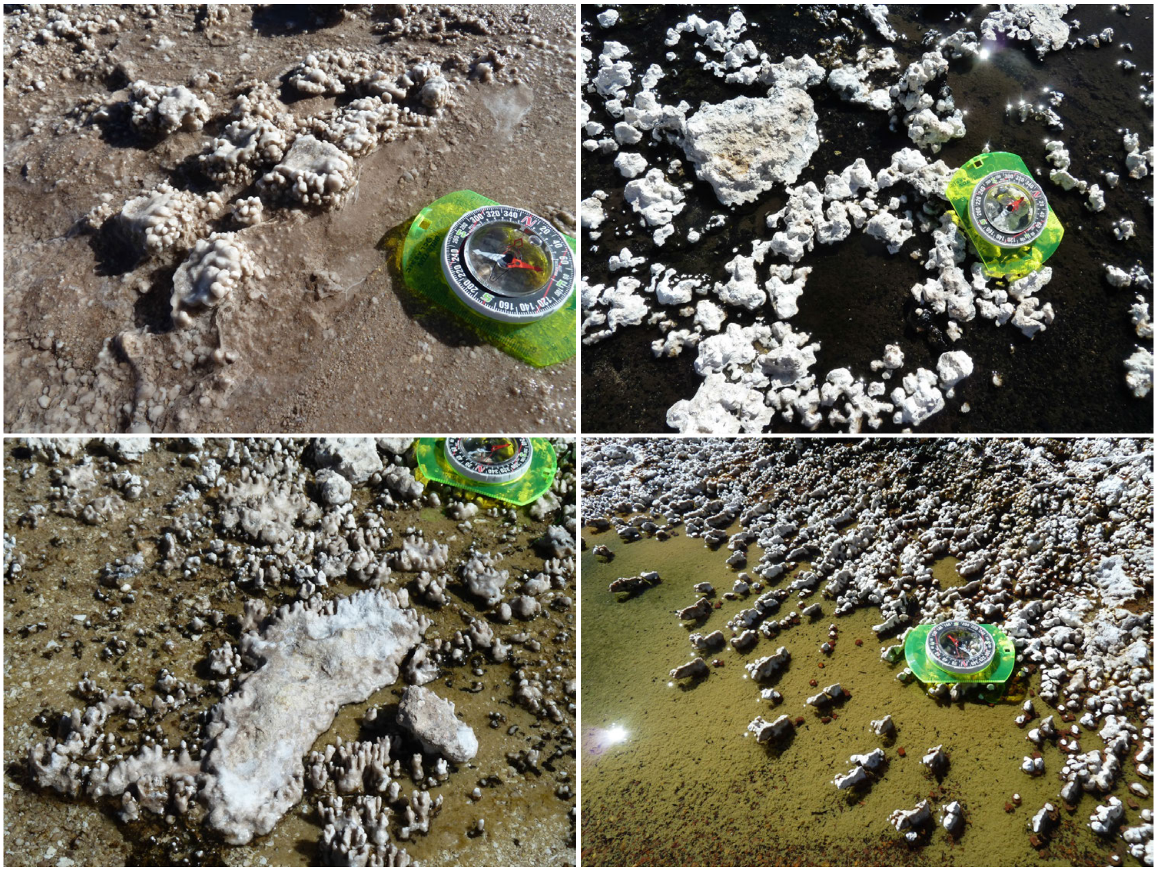

Supplementary Figure 2 | Examples of the diverse morphology of opaline silica nodular and digitate structures in hot spring and geyser outflow channels at El Tatio. Straight edge of compass is ~6 cm long.

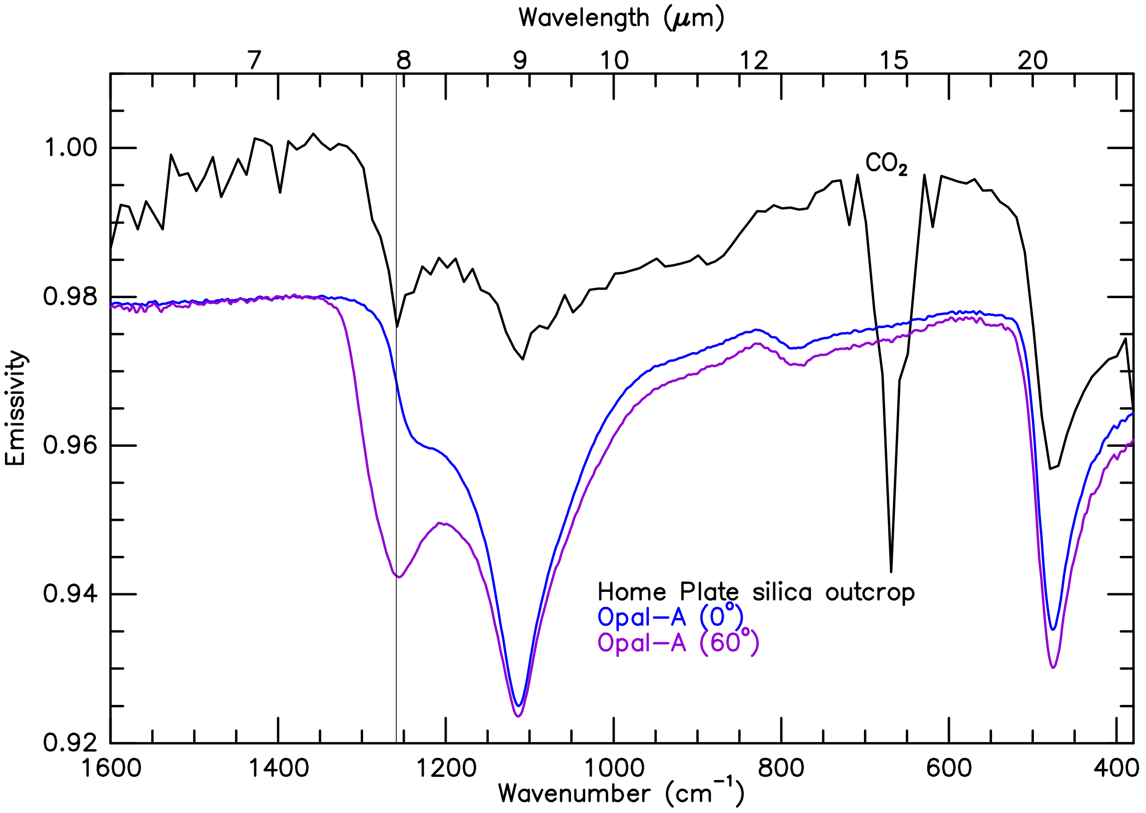

Supplementary Figure 3 | Comparison of a Mini-TES spectrum of Home Plate silica outcrop to laboratory spectra of opal-A measured at different emission angles. A feature near 1260 cm^{-1} (vertical line) in the Mini-TES spectrum (black) is not present in opal-A measured at an emission angle of 0° (blue spectrum). This feature is present when opal-A is measured at high emission angle (60°; purple spectrum). The Mini-TES spectrum is from the target Clara Zaph4, sol 1168, P3968. Both opal-A spectra have been contrast reduced by 90% and offset.

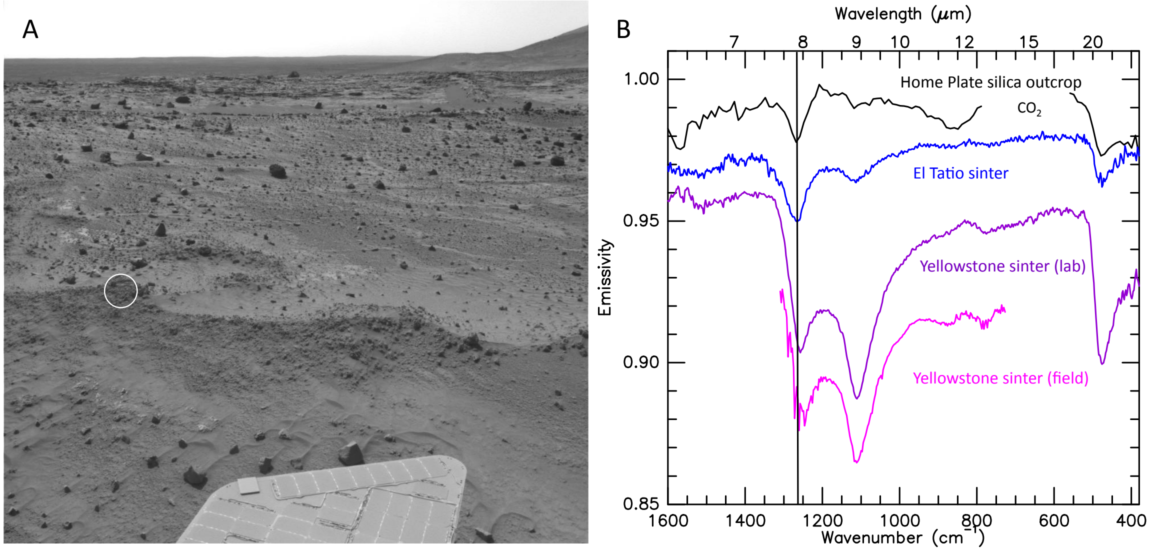

Supplementary Figure 4 | Example of Home Plate opaline silica nodular outcrop with distinctive spectral characteristics attributable to halite. (A) Mini-TES observed the "Kobal" target (white circle ~20 cm in diameter; sol 1116, P3857) on a portion of the same outcrop shown in Fig. 1 but viewed from the east toward Home Plate in this Navcam frame (sol 1114, P0775). **(B)** A very strong feature near ~1260 cm^{-1} (vertical line) with a weak feature near 1100 cm^{-1} in the Mini-TES spectrum (black; scaled by 2x) is matched by the spectrum of a halite-encrusted El Tatio sinter sample (blue; offset), but not halite-free sinter from Yellowstone measured at a high emission angle (>60°) in the laboratory (purple; offset) and field (magenta; offset). Broad feature centered near 850 cm^{-1} could be due to surface dust.

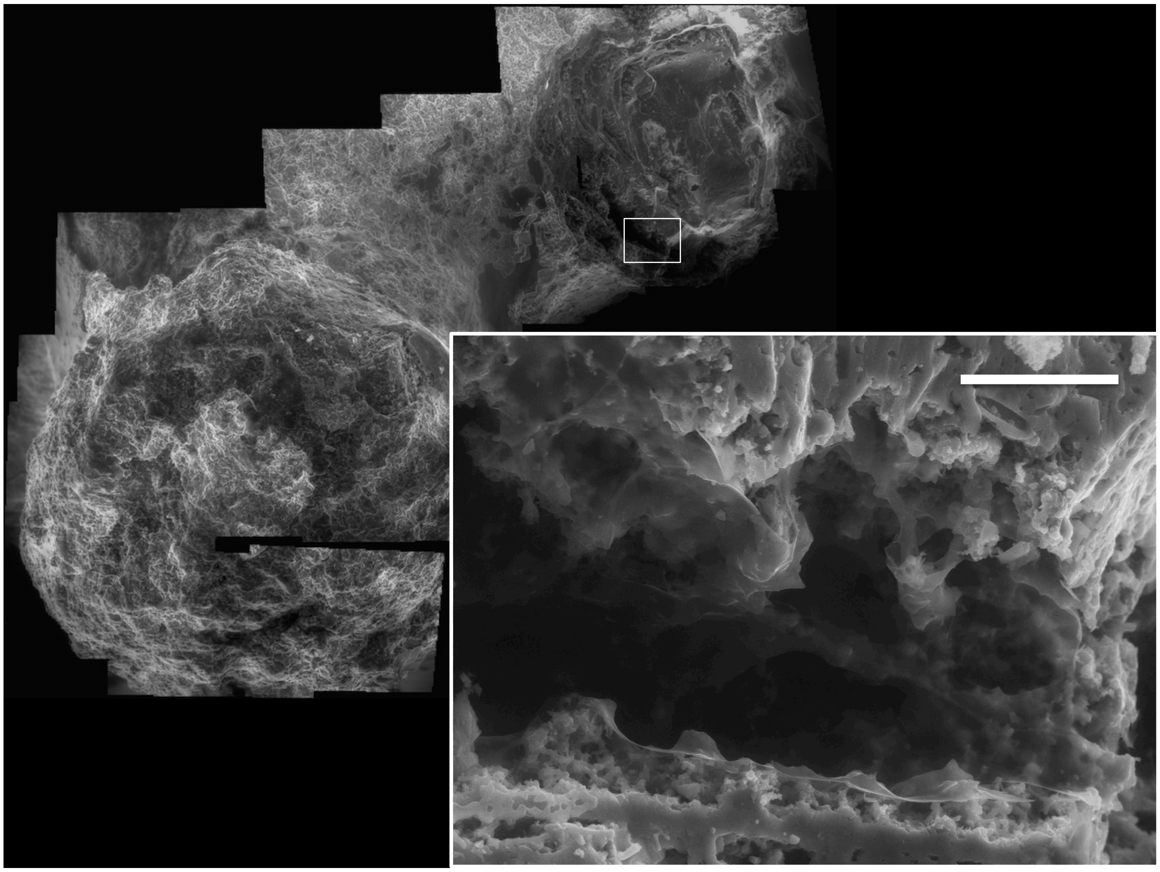

Supplementary Figure 5 | Environmental SEM views of El Tatio digitate silica structures. Background mosaic of ESEM images (sample ET3-3B) shows a protruding structure at left and the base of another structure after it was broken off in the upper right. The white box represents the enlarged inset, which shows a large fenestral cavity in which a film of electron translucent (25 kV) material that we interpret as unsilicified microbial exopolymeric substance drapes filamentous sinter. The white scalebar represents 50 μm.

Supplementary Table 1 | Semi-quantitative elemental data produced from energy dispersive spectroscopy of El Tatio sinter sample ET3-3A before and after scrubbing with a toothbrush and deionized water. As shown in Fig. 5c and 5d, the abundance of Na and Cl diminished and Si and O increased following scrubbing, indicative of a loss of halite. The substantial abundance of C likely is due to surficial organic matter.

Element	Atomic % (before)	Atomic % (after)
C	32.72	16.89
O	39.17	55.12
Na	8.11	2.54
Si	12.23	21.99
Cl	6.81	2.12
K	ND	0.45
Ca	2.11	0.88
